# Elevated Levels of Circulating Biomarkers Related to Leaky Gut Syndrome and Bacterial Translocation Are Associated With Graves’ Disease

**DOI:** 10.3389/fendo.2021.796212

**Published:** 2021-12-16

**Authors:** Dekai Zheng, Huimin Liao, Shuze Chen, Xiuying Liu, Chuyin Mao, Cangui Zhang, Min Meng, Zhi Wang, Ying Wang, Qinrui Jiang, Yaoming Xue, Lin Zhou, Ye Chen

**Affiliations:** ^1^ Guangdong Provincial Key Laboratory of Gastroenterology, State Key Laboratory of Organ Failure Research, Department of Gastroenterology, Nanfang Hospital, Southern Medical University, Guangzhou, China; ^2^ Integrative Clinical Center of Microecology, Department of Gastroenterology, Shenzhen Hospital, Southern Medical University, Guangzhou, China; ^3^ Department of Endocrinology and Metabolism, Nanfang Hospital, Southern Medical University, Guangzhou, China

**Keywords:** intestinal barrier, leaky gut, bacterial translocation, Graves’ disease, lipopolysaccharide, intestinal fatty acid-binding protein, quality of Life

## Abstract

**Background:**

A growing number of studies have found dysbiosis of the intestinal microbiota in patients with Graves’ disease (GD). The intestinal epithelial barrier serves as the first line of defense, protecting the immune system from excessive stimulation of microbiota and toxins. Most autoimmune diseases are associated with a gut barrier dysfunction (leaky gut) which allows bacterial translocation. However, to date, potential correlations between intestinal barrier dysfunction and GD have not been explored.

**Methods:**

Serum lipopolysaccharide (LPS), intestinal fatty acid-binding protein (I-FABP), zonulin, D-lactate, and diamine oxidase (DAO) were measured to assess barrier integrity in 91 patients with GD (61 initial GD and 30 euthyroid GD) and 44 healthy controls. The quality of life (QOL) of patients with GD was assessed using the thyroid-specific patient-reported outcome (ThyPRO-39) questionnaire.

**Results:**

The serum levels of LPS, I-FABP, zonulin, and D-lactate were significantly higher in patients with initial GD than in healthy controls. Logistic regression analysis revealed that zonulin and D-lactate were independently associated with risk for GD and circulating zonulin could effectively distinguish patients with initial GD from healthy controls. Correlation analyses showed that I-FABP, LPS, and D-lactate were positively associated with FT4 and negatively associated with TSH. In addition, circulating LPS, zonulin, and D-lactate levels were all independent predictors of TRAb levels. Moreover, higher circulating LPS levels in patients with GD were associated with more severe hyperthyroidism (higher concentrations of FT3, FT4, and TRAb and lower TSH concentrations) and worse scores of hyperthyroid and eye symptoms.

**Conclusion:**

Patients with initial GD show a disrupted intestinal barrier, characterized by elevated levels of leaky gut biomarkers. Increased intestinal permeability and bacterial translocation were associated with TRAb levels and hyperthyroidism in GD. Further research is required to elucidate the underlying mechanisms.

## Introduction

Graves’ disease (GD) is an organ-specific autoimmune disorder characterized by excessive secretion of thyroid hormones and the presence of serum antithyroid antibodies, especially thyrotropin receptor autoantibodies (TRAb). It is the most common cause of hyperthyroidism, affecting approximately 2% of women and 0.2% of men (1, 2), and is associated with increased susceptibility to multiple autoimmune disorders (3). Moreover, a prospective cohort study has shown that patients with GD still experience impaired quality of life (QOL) even after drug treatment (4). Although it is commonly accepted that the excessive production of TRAb induced by an abnormal immune response is an important cause of the development of GD, the specific pathogenesis of GD remains unclear.

In recent years, the relationship among the intestinal barrier, microbiota, and the immune system has received increasing attention. The intestinal tract is the largest immune organ in the human body and is colonized by nearly 100 trillion microorganisms, known as the microbiota (5). Under physiological conditions, the intestinal microbiota play a key role in maintaining immune homeostasis and improving the development of the immune system through the direct influence of their components and metabolites on immune cells (6, 7). Multiple studies have shown an apparent alteration of gut microbiota composition, called dysbiosis, in patients with GD, characterized by significantly reduced diversity of microbiota, decreased commensal microbes, and lower levels of beneficial bacterial metabolites (8, 9). The intestinal epithelial barrier is composed of a continuous single layer of epithelial cells joined by tight junction proteins which seal paracellular route. They act as a selectively permeable barrier that allows the absorption of water, electrolytes, and nutrients while preventing the passage of antigens, intestinal microbiota, and toxins (10). Microbiota dysregulation may disrupt the epithelial barrier and lead to increased intestinal permeability, allowing bacteria and their products to translocate into the circulation and resulting in persistent immune activation (11). Gut barrier dysfunction, also known as “leaky gut”, has been associated with multiple autoimmune diseases and chronic inflammatory disorders, including type 1 diabetes, systemic lupus erythematosus, multiple sclerosis, celiac disease, and inflammatory bowel diseases (10, 12). Moreover, according to the gut-thyroid axis hypothesis, bacterial translocation resulting from increased intestinal permeability may stimulate the immune system toward a pro-inflammatory state, thereby further affecting thyroid function and promoting the development of autoimmune thyroid diseases (13). Given these facts, we speculated that leaky gut syndrome might be associated with the pathogenesis of GD. However, to date, no studies have explored the association between leaky gut and GD.

Several circulating biomarkers have been tested and validated for the assessment of intestinal barrier integrity and bacterial translocation. Intestinal fatty acid-binding protein (I-FABP) and diamine oxidase (DAO) are cytosolic proteins in intestinal epithelial cells and are immediately released into the circulation when the intestinal epithelium is disrupted (14, 15). Zonulin, the only known physiological regulator of intestinal permeability, reversibly opens tight junctions between intestinal epithelial cells (16). In addition, lipopolysaccharide (LPS) and D-lactate can function as sensitive indicators of the translocation of gut-resident bacteria and their metabolites (17, 18).

The current study aimed to investigate the changes in gut barrier function in patients with GD and explore whether increased intestinal permeability and bacterial translocation into the circulation are associated with hyperthyroidism and higher QOL survey scores in patients with GD.

## Participants and Methods

### Participants

This cross-sectional study was conducted at the Nanfang Hospital of Southern Medical University, Guangzhou, China. A total of 91 patients with GD (61 initial GD and 30 euthyroid GD) were enrolled. Initial GD was newly diagnosed according to the American Thyroid Association Guidelines (19): 1) typical clinical manifestations of hyperthyroidism, such as heat sensitivity, moist skin, weight loss, anxiety, irritability, etc.; 2) elevated thyroid hormone levels (FT3 and FT4) and suppressed serum thyroid-stimulating hormone (TSH) in the presence of thyrotropin receptor antibody (TRAb); and 3) diffuse goiter and increased vascularity in the thyroid. Euthyroid GD was defined as patients with GD with normal FT3, FT4, and TSH levels after treatment with antithyroid drugs. In addition, 44 healthy participants served as a control group. These individuals had no medical or family history of GD. The exclusion criteria included pregnancy, infections, cancer, other autoimmune diseases, antibiotics, or probiotic use within 3 months of enrollment, as well as a history of gastrointestinal surgery or malignancy.

Ethical approval was obtained from the Institutional Review Board of Nanfang Hospital of Southern Medical University. All participants completed informed consent forms.

### Data Collection

Demographic information was collected through face-to-face interviews using pre-constructed questionnaires. To assess the patients’ QOL, patients with GD completed the thyroid-specific patient-reported outcome (ThyPRO-39) questionnaire (20). The average score of items on each scale is divided by four and multiplied by 100 to yield scales from 0 to 100, with higher scores indicating worse QOL.

### Measurement of Gut Barrier Biomarkers

All participants were required to fast for at least 12 h before blood collection. Approximately 2 mL of blood was centrifuged at 2500 g for 10 min. Serum was collected and stored at −80°C prior to analysis. Serum concentrations of intestinal barrier markers (LPS, I-FABP, zonulin, and DAO) were determined using enzyme-linked immunosorbent assay kits (CUSABIO, Wuhan, China) according to the manufacturer’s instructions. The intra-assay and inter-assay coefficients of variation were <8% and <10%, respectively. D-lactate concentrations were measured using a colorimetric assay kit (Elabscience, Wuhan, China) according to the manufacturer’s instructions. The intra-assay and inter-assay precisions were 3.8% and 7.7%, respectively.

### Statistical Analysis

All statistical analyses were conducted using SPSS version 20.0 (IBM Corp., Armonk, NY, USA). The distributions of variables were assessed using the Shapiro–Wilk test. Data are expressed as means ± standard deviations (continuous variables with a normal distribution) or medians with interquartile ranges (continuous variables with a non-normal distribution) and as counts (%) for categorical data. The study groups were compared using Student’s t-test, one-way analysis of variance, Mann–Whitney U test, Kruskal–Wallis test, or chi-square test. Spearman’s rank test and Pearson’s correlation coefficient were used to analyze correlations. Multiple linear regression was used to identify the factors associated with TRAb levels. Multivariable logistic regression was conducted to investigate the associations between circulating leaky gut biomarkers and GD. Receiver operating characteristic (ROC) curves were constructed to evaluate the diagnostic values of these biomarkers. Statistical significance was set at *p<*0.05 (two-sided).

## Results

### Demographic and Clinical Characteristics of Participants

**Table 1** summarizes the demographic and clinical characteristics of all participants. Patients with initial GD presented a significant increase in serum platelets (PLT) (273.0 ± 62.0 vs. 238.0 ± 62.0), alanine transaminase (ALT) [27 (17.5-39.0) vs. 10.5 (8.0-12.7)], and aspartate aminotransferase (AST) [22 (17.0-32.0) vs. 15.0 (13.2-17.0)] levels relative to healthy controls, while ALT [27 (17.5-39.0) vs. 16.0 (10.5-22.5)] and AST [22 (17.0-32.0) vs. 17.5 (14.0-20.6)] were significantly decreased in patients with euthyroid GD, compared to those with initial GD. In addition, patients with initial GD showed increased levels of FT3, FT4, TRAb and increased scores of hyperthyroid symptoms, goiter symptoms, eye symptoms, tiredness, and more severe daily life impairments compared to those with euthyroid GD (all *p*<0.001).

**Table 1:** Demographic and clinical characteristics of patients with GD and control participants.

Variables	Control group	Initial GD	Euthyroid GD
Sample size	44	61	30
Age (years)	29.3 ± 8.2	33.6 ± 11.3	32.4 ± 9.1
Female n (%)	31 (70)	43 (70)	25 (83)
BMI (kg/m²)	21.0 ± 2.6	20.4 ± 2.3	21.4 ± 3.0
WBC (10^9^/L)	6.0 ± 1.3	6.2 ± 1.4	6.5 ± 1.6
LYM (10^9^/L)	2.1 ± 0.4	2.3 ± 0.5	2.3 ± 0.6
NEU (10^9^/L)	3.3 ± 0.9	3.2 ± 1.2	3.6 ± 1.0
HGB (g/L)	134.3 ± 15.8	142.0 ± 16.8	137.3 ± 15.5
PLT (10^9^/L)	238.0 ± 62.0	273.0 ± 62.0*	282.5 ± 83.4*
ALT (U/L)	10.5 (8.0-12.7)	27 (17.5 + 39.0) **	16.0 (10.5-22.5) ^##^
AST (U/L)	15.0 (13.2-17.0)	22 (17.0-32.0) **	17.5 (14.0-20.6) ^##^
TBIL (μmol/L)	12.9 (9.2-16.1)	11.6 (9.2-16.2)	9.0 (7.5-12.8)
DBIL (μmol/L)	4.1 (3.4-5.8)	4.4 (3.5-5.6)	4.2 (2.6-4.5)
TGAb (IU/ml)	–	183.4 (21.8 -687.3)	292.1 (35.9-743.4)
TPOAb (IU/ml)	–	150.0 (46.9-369.7)	194.9 (57.4-301.9)
TRAb (IU/L)	–	14.9 ± 12.1	2.6 ± 3.2^##^
FT3 (pmol/L)	–	13.2 ± 5.3	3.0+0.4^##^
FT4 (pmol/L)	–	49.5 (34.3-67.8)	14.6 (13.0-16.6) ^##^
TSH (mIU/L)	–	0.004 (0.003-0.007)	1.9 (0.8-2.9) ^##^
LPS (pg/ml)	169.8 (131.9-196.6)	178.5(142.9-275.8) *	106.7 (89.1-189.2) ^##^
I-FABP (ng/ml)	4.77(4.67-4.84)	4.97(4.70-5.53) **	4.80(4.59-4.96) ^#^
Zonulin (ng/ml)	6.78(5.9-7.7)	11.3(9.8-13.9) **	9.4(7.8-12.8) ^#^
D-lactate (mmol/L)	0.57 ± 0.03	0.75 ± 0.03**	0.57 ± 0.05^#^
DAO (mIU/ml)	115(100.8-148.7)	107.8(87.4-138.5)	90.4(76.9-108.3) ^#^
**Quality of life**			
Hyperthyroid symptoms	–	41.6 (25.0-68.7)	6.2 (0-18.7) ^##^
Hypothyroid symptoms	–	12.5 (6.2-18.7)	9.3 (0-18.7)
Goiter symptoms	–	8.3 (0-25.0)	0 (0-8.3) ^##^
Eye symptoms	–	16.6 (8.3-41.6)	8.3 (0-10.4) ^##^
Tiredness	–	41.6 (25-50)	25 (16.6-33.3) ^##^
Cognitive complaints	–	16.6 (8.3-25)	8.3 (0-25)
Anxiety	–	25 (0-25)	16.6 (0-25)
Depressivity	–	16.6 (8.3-33.3)	16.6 (8.3-33.3)
Emotional susceptibility	–	33.3 (16.6-50)	25 (16.6-33.3)
Impaired social life	–	16.6 (0-25)	0 (0-16.6)
Impaired daily life	–	8.3 (0-8.3)	0 (0-0) ^##^
Cosmetic complaints	–	16.6 (0-25)	0 (0-16.6)

Data are expressed as mean ± standard deviation or median (interquartile range).

*p < 0.05, **p < 0.01versus control group, ^#^p < 0.05, ^##^p < 0.01 versus initial GD.

“-” indicates that the parameter was not detected.

BMI, body mass index; WBC, white blood cell; LYM, lymphocyte; NEU, neutrophils; HGB, hemoglobulin; PLT, platelet; ALT, alanine aminotransferase; AST, aspartate aminotransferase; TBIL, total bilirubin; DBIL, direct bilirubin; FT3, free triiodothyronine; FT4, free thyroxine; TSH, thyroid-stimulating hormone; TRAb, thyrotropin receptor antibody. TPOAb, thyroid peroxide antibody; TGAb, thyroglobulin antibody.

Normal reference range: TPOAb (0.00-34.00 IU/mL), TgAb (0.00-115.00 IU/mL), TRAb (0.000-1.750 IU/L), FT3 (3.54-6.47 pmol/L), FT4 (11.57-22.88 pmol/L), and TSH (0.55-4.78 mIU/L).

### Increased Intestinal Permeability Independently Associated With Risk for GD and Zonulin as a Biomarker for the Diagnosis of GD

**Figure 1:**
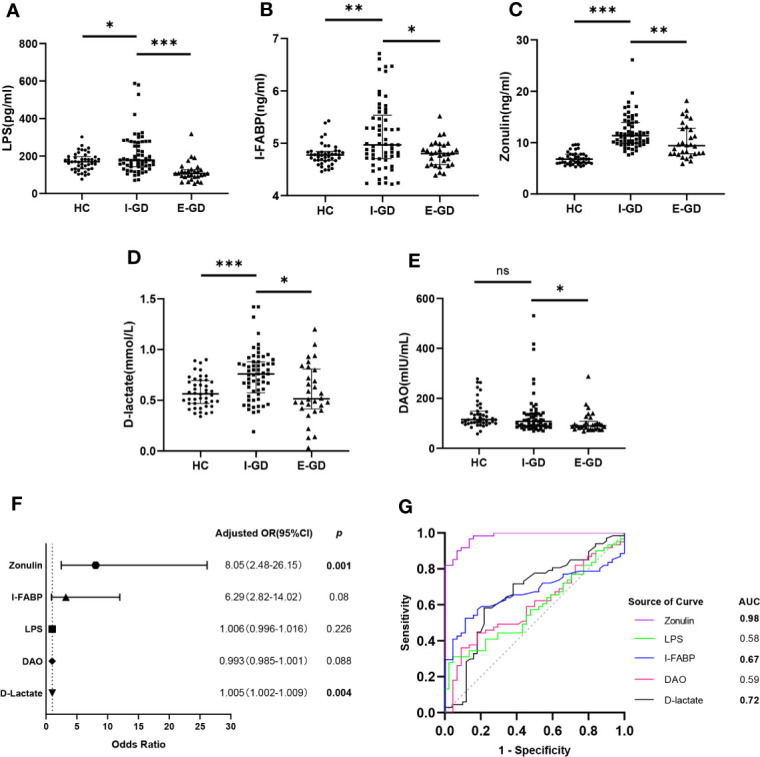
Increased intestinal permeability independently associated with risk for GD and zonulin as a biomarker for the diagnosis of GD. Comparison of serum levels of LPS **(A)**, I-FABP **(B),** zonulin **(C)**, D-lactate **(D)** and DAO **(E)** between HC and GD; **(F)** Forest plot of circulating leaky gut biomarkers associated with GD risk; **(G)** ROC analysis of circulating leaky gut biomarkers for GD diagnosis; Bars represent the mean ± SD. *p < 0.05; **p < 0.01; ***p < 0.001; LPS, lipopolysaccharides; I-FABP, intestinal fatty acid binding protein; DAO, diamine oxidase; HC, healthy controls; GD, Graves’ disease.

The serum levels of LPS, I-FABP, zonulin, and D-lactate were significantly higher in patients with initial GD than the levels of those molecules in the control group (**Figures 1A–D**), whereas no difference in DAO levels was found between the two groups (**Figure 1E**). Interestingly, these biomarkers were significantly decreased in patients with euthyroid GD relative to those with initial GD (**Figures 1A–E**). Multivariate logistic regression analysis showed that zonulin (adjusted OR=8.05, *p*=0.001) and D-lactate (adjusted OR=1.005, *p*=0.004) were risk factors for GD, even after controlling for age, sex, BMI, PLT, ALT, and AST (**Figure 1F**). Moreover, we evaluated the clinical usefulness of leaky gut biomarkers for initial GD diagnosis using ROC curve analyses. Zonulin yielded the largest area under the ROC curve (AUC=0.98, *p*<0.0001) (**Figure 1G**). The optimal cut-off value for zonulin for discriminating between patients with initial GD and healthy individuals was 8.9 ng/mL (sensitivity=90.2%, specificity=93.2%).

### Leaky Gut Biomarkers Associated With Thyroid Function, Laboratory Parameters, and QOL in Patients with GD

**Figure 2:**
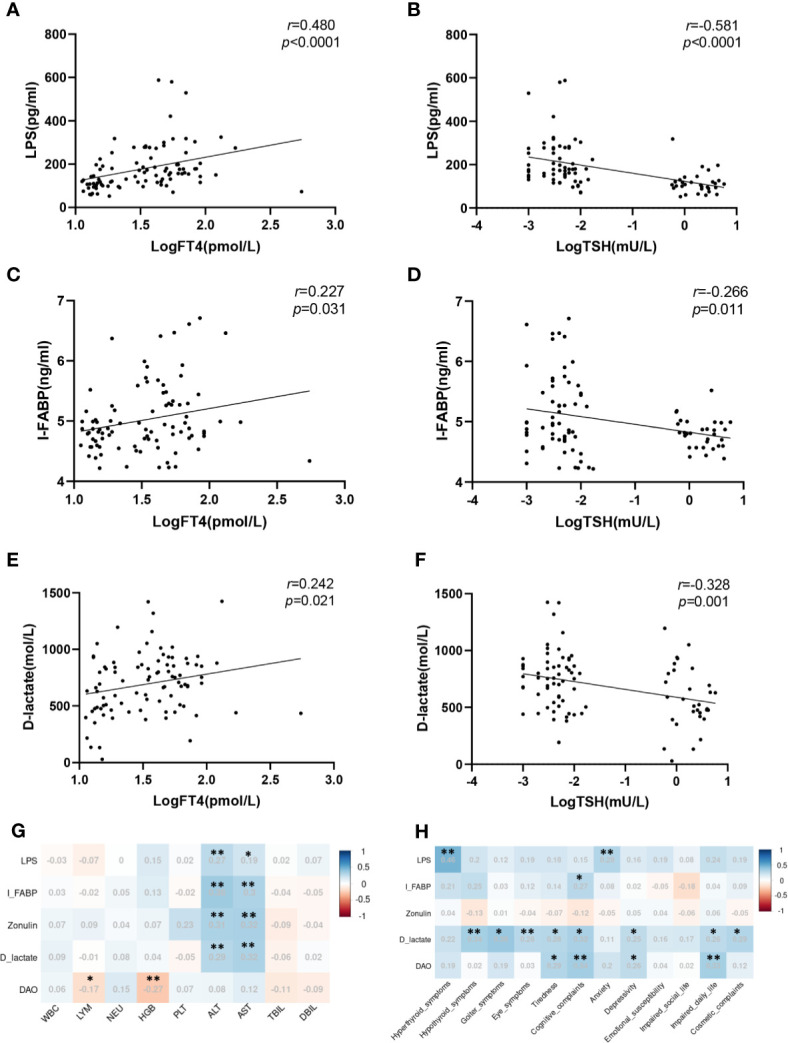
Leaky gut biomarkers associated with thyroid function, laboratory parameters, and quality of life in patients with GD. **(A)** Correlation of serum LPS with log-transformed FT4; **(B)** Correlation of serum LPS with log-transformed TSH; **(C)** Correlation of serum I-FABP with log-transformed FT4; **(D)** Correlation of serum I-FABP with log-transformed TSH; **(E)** Correlation of serum D-lactate with log-transformed FT4; **(F)** Correlation of serum D-lactate with log-transformed TSH; **(G)** Correlation of circulating gut barrier biomarkers with laboratory parameters; **(H)** Correlation of circulating gut barrier biomarkers with quality of life; *p < 0.05; **p < 0.01; FT4, free thyroxine; TSH, thyroid stimulating hormone.

Furthermore, we analyzed the correlation between leaky gut biomarkers and thyroid function. Serum LPS, I-FABP, and D-lactate levels were significantly positively correlated with FT4 levels (*r*=0.480, *p*<0.0001; *r*=0.227, *p*=0.031; *r*=0.242, *p*=0.021) and negatively correlated with TSH (*r*=-0.581, *p*<0.0001; *r*=-0.266, *p*=0.011; *r*=-0.328, *p*=-0.001) (**Figures 2A–F**). In addition, we found that, except for DAO, all leaky gut biomarkers positively correlated with ALT and AST (**Figure 2G**). Furthermore, LPS was significantly positively correlated with scores for hyperthyroid symptoms (r=0.46, *p<*0.001) and anxiety (r=0.29, *p=*0.022), whereas I-FABP was positively correlated with cognitive complaint scores (r=0.27, *p=*0.034). Surprisingly, a significant positive association between D-lactate and most QOL-related indicators was found (all p*<*0.05). And DAO was positively associated with tiredness (r=0.27, *p=*0.034), cognitive complaints (r=0.34, *p=*0.006), depressive disorder (r=0.26, *p=*0.045) and impaired daily life (r=0.33, *p=*0.009). No association was found between zonulin and QOL in patients with GD (**Figure 2H**).

### Leaky Gut Biomarkers Especially LPS Associated With TRAb Levels in GD

**Figure 3:**
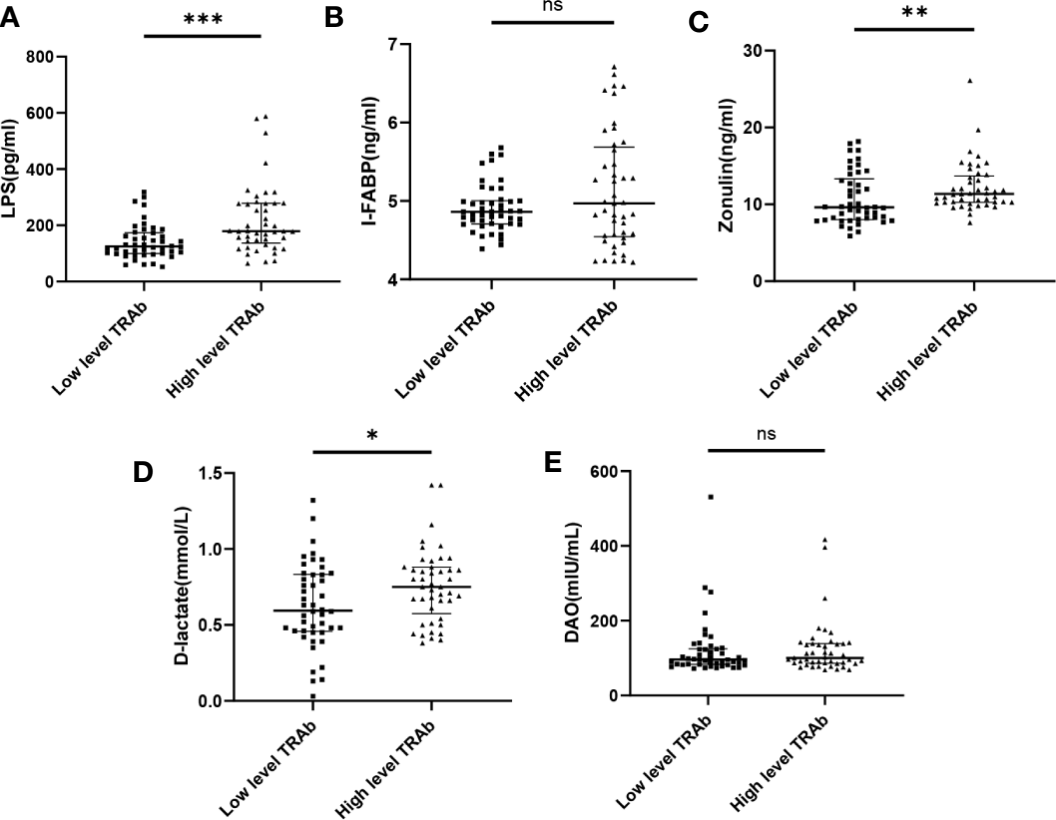
Leaky gut biomarkers associated with TRAb levels in GD. Comparison of serum levels of LPS **(A)**, I-FABP **(B),** zonulin **(C)**, D-lactate **(D)** and DAO **(E)** in patients with GD between low and high TRAb subgroup. Bars represent the mean ± SD. *p < 0.05; **p < 0.01; ***p < 0.001. "ns" indicates not significant.

To explore the association between leaky gut syndrome and TRAb levels, patients with GD were divided into high and low-level TRAb subgroups according to the median of their serum TRAb concentrations. Patients with GD with high-level TRAb had higher levels of LPS, I-FABP, zonulin, and D-lactate, but not DAO, compared to those with low-level TRAb (all *p*<0.05) (**Figures 3A–E**). Multiple linear regression revealed that LPS, zonulin, and D-lactate were independently associated with TRAb (*β*=0.985, *p*<0.0001; *β*=1.453, *p*=0.003; *β*=0.001, *p*=0.009) (**Table 2**). Finally, to further explore the relationship between LPS and hyperthyroidism of GD, patients with GD were divided into a high-LPS and a low-LPS subgroup according to the median of their serum LPS concentrations. As shown in **Table 3**, GD patients with high-LPS levels had lower TSH levels (*p<*0.001), higher concentrations of FT3 (*p<*0.001), FT4 (*p<*0.001), and TRAb (*p=*0.003); higher scores for hyperthyroid (*p<*0.001) and eye symptoms (*p=*0.004); and more severe impairments of daily life (*p=*0.003), than those with low-LPS levels.

**Table 2:** Multiple linear regression model of variables associated with serum TRAb.

Variable	Unadjusted			Adjusted		
	*β*	95.0%CI (*β*)	*p*-value	*β*	95.0%CI (*β*)	*p*-value
LPS	1.027	0.534-1.520	**<0.0001**	0.985	0.467-1.503	**<0.0001**
I-FABP	1.616	-0.905-4.137	**0.206**	1.467	-1.037-3.772	0.118
Zonulin	1.342	0.383-2.301	**0.007**	1.453	0.519-2.387	**0.003**
D-lactate	0.001	0-0.001	**0.018**	0.001	0-0.001	**0.009**
DAO	0	-0.001-0.002	0.892	0	-0.001-0.002	0.798

The values of TRAb, LPS, I-FABP and zonulin were Log-transformed.

Adjusted for age, gender, BMI.

Statistically p values are bolded.

**Table 3:** Comparison of the thyroid function and quality of life in patients with GD between low and high LPS subgroup.

Variables	Low level LPS	High level LPS	*p*
Sample size	45	46	
LPS	110.2 (93.0-128.1)	204.6 (177.2-284.2)	**<0.001**
**Thyroid function**			
TGAb (IU/ml)	337.4 (21.9-582.6)	162.0 (14.7-677.9)	0.275
TPOAb (IU/ml)	193.3 (76.4-294.2)	274.1 (50.5-474.0)	0.959
TRAb (IU/L)	7.2 ± 10.0	14.4 ± 12.1	**0.003**
FT3 (pmol/L)	7.1 ± 5.9	12.6 ± 5.8	**<0.001**
FT4 (pmol/L)	15.4 (13.2-24.3)	47.5 (28.1-62.9)	**<0.001**
TSH (mIU/L)	0.99 (0.014-2.541)	0.005 (0.003-0.008)	**<0.001**
**Quality of life**			
Hyperthyroid symptoms	12.5 (0-25)	39.5 (24.4-64.0)	**<0.001**
Hypothyroid symptoms	12.5 (6.2-18.7)	16.6 (6.2-25)	0.135
Goiter symptoms	0 (0-8.3)	8.3 (0-10.4)	0.289
Eye symptoms	8.3 (0-16.6)	25 (8.3-41.6)	**0.004**
Tiredness	33.3 (16.6-41.6)	41.6 (25-50)	0.142
Cognitive complaints	8.3 (0-25)	16.6 (8.3-25)	0.426
Anxiety	16.7 (0-25)	16.6 (6.2-25)	0.430
Depressivity	16.6 (8.3-33.3)	16.6 (8.3-33.3)	0.505
Emotional susceptibility	25 (16.6-33.3)	29.1 (14.5-52)	0.289
Impaired social life	8.3 (0-16.6)	8.3 (0-25)	0.829
Impaired daily life	0	8.3 (0-10.4)	**0.003**
Cosmetic complaints	8.3 (0-16.6)	8.3 (0-25)	0.658

Significant p values are bolded.

## Discussion

This study, which is the first to investigate correlations between circulating leaky gut biomarker levels and GD, reveals that increased intestinal permeability and bacterial translocation are associated with impaired thyroid function and decreased QOL in patients with GD.

As the first line of defense, the intestinal epithelial barrier physically separates luminal contents from the internal milieu and protects the immune system from excessive stimulation by microbiota and toxins. Disruption of the epithelial barrier leads to increased intestinal permeability, which allows bacteria and their products to translocate into the circulation, entailing persistent immune activation (11). In line with this observation, a previous study has shown that bacterial translocation contributes to type 1 diabetes development by activating the nucleotide-binding oligomerization domain-containing protein 2 (NOD2) inside the pancreatic lymph nodes (21). Furthermore, in the systemic lupus erythematosus mouse model, translocation of *Enterococcus gallinarum from the gut to the liver* drives autoimmune pathogenesis by skewing T helper cell differentiation and directly inducing autoantigens (22). Indeed, increased intestinal permeability favoring bacterial translocation has been considered a danger signal for autoimmune diseases as it can enhance the production of autoantibodies through exposure to foreign antigens (10, 23). As multiple studies have confirmed the presence of microbiota dysregulation in patients with GD, it is of great significance to explore the alteration of intestinal barrier integrity and bacterial translocation in this disease. In the present study, we observed that leaky gut biomarkers were significantly elevated in the serum of patients with initial GD and increasing levels of zonulin and D-lactate were independently associated with a higher risk of the disorder. These results indicate that gut barrier disruption and bacterial translocation may be involved in the development of GD.

High levels of serum I-FABP and zonulin in patients with GD indicate intestinal epithelial cell injury and the opening of tight junctions between intestinal epithelium, which lead to increased intestinal permeability. Dysregulated microbiota in patients with GD can damage intestinal epithelial cells through direct contact, toxin release, and activation of innate immunity, which may be a reason for increased levels of I-FABP and zonulin (11). In addition, dietary gluten also induces zonulin release by binding to the CXCR3 receptor, which has been confirmed to participate in the pathogenesis of celiac disease (24, 25). Of note, a pilot study showed that following a gluten-free diet for six months significantly reduced the serum titers of thyroid peroxidase antibodies and thyroglobulin antibodies in patients with Hashimoto’s thyroiditis (26). This manifestation suggests that elevated levels of zonulin induced by gluten and pathogenic microbiota may be implicated in the pathogenesis of GD. Furthermore, zonulin levels exhibited sufficient sensitivity and specificity to distinguish patients with initial GD from healthy individuals. Therefore, we suggest that testing serum zonulin levels could assist in the diagnosis of GD. However, further large-scale studies to evaluate the clinical value of serum zonulin are essential to assess its impact on the diagnosis of GD.

In addition, we found that the levels of the biomarkers of intestinal leakage in patients with euthyroid GD were significantly lower than in patients with initial GD. Further correlation analysis showed that LPS, I-FABP, and D-lactate were positively correlated with FT4 and negatively correlated with TSH. TRAb is the main pathogenic autoantibody involved in the hyperthyroidism disorder associated with GD. As self-tolerance against the TSH receptor is lost, thyroid B cells differentiate into plasma cells and release TRAb (27). Our study shows that zonulin, LPS and D-lactate were independently associated with the level of TRAb in patients with GD. Moreover, the high-LPS subgroup exhibited higher TRAb levels and more severe hyperthyroidism compared to the low-LPS subgroup. Thus, high levels of LPS, and D-lactate suggest an increase in the translocation of bacteria and their metabolites, which may contribute to the pathogenesis of GD in multiple ways. On the one hand, *in silico* analyses have shown that some antigens from microbes such as *Candida albicans*, *Helicobacter pylori* and, *Lactobacilli* can directly trigger thyroid autoimmunity *via* molecular mimicry (28). On the other hand, a pro-inflammatory status has also been associated with an elevated risk of GD (29). It is well established that circulating LPS has the potential to cause a pro-inflammatory status by activating Toll-like receptor 4 (TLR4) and initiating NF-κB signaling pathways (30). For instance, CD14 is a receptor for LPS, whose single nucleotide polymorphisms were associated with higher genetic susceptibility for GD (31). In patients with type 2 diabetes, higher LPS levels are positively correlated with inflammatory factors such as TNF-α and IL-6 (32). Overall, our findings support the theory that bacterial translocation plays an essential role in the pathogenesis of GD. Additional *in vitro* and *in vivo* studies are required to uncover the underlying mechanism.

Furthermore, most leaky gut biomarkers were positively associated with high levels of ALT and AST. As markers for evaluating liver function, serum levels of both ALT and AST were increased in patients with initial GD compared with healthy controls, according to a previous report by Li et al. (33). Moreover, correlation analysis revealed that in addition to thyroid-related symptoms, leaky gut biomarkers were also positively correlated with extra-thyroid symptoms such as eye symptoms, tiredness, depressive disorders, and impaired daily life quality in patients with GD. Thus, we speculate that translocated bacteria and their metabolites may reach extra-thyroid organs through the bloodstream, activate innate immunity, and ultimately affect the QOL and liver function of patients with GD. It has recently been reported that enhanced gut LPS translocation into the bloodstream mediates antithyroid drug-induced liver injury (34). In addition, several studies have confirmed that circulating LPS promotes neuroinflammation by inducing oxidative stress (35) and activation of microglia (36). Increased intestinal permeability and bacterial translocation have been associated with a growing number of neuropsychiatric and hepatic disorders (37–39). Thus, it is necessary to pay closer attention to the potential effect of leaky gut on liver function and QOL in patients with GD, and the maintenance of intestinal barrier function may contribute to improving the prognosis of such patients.

While these findings are novel, the present study has several limitations that need to be mentioned. First, a definitive causal relationship between intestinal barrier function and GD could not be established because of the cross-sectional design. Therefore, further prospective studies are required to confirm these findings. Notably, increased levels of leaky gut biomarkers in patients with initial GD, but not euthyroid GD, suggest that these changes may be due to an increase in the levels of thyroid hormones. To confirm the causality, additional studies on patients with non-autoimmune hyperthyroidism, such as toxic adenoma, will be necessary. Second, only five commonly used leaky gut biomarkers were identified in our study, which may have led to an incomplete assessment of intestinal barrier function and bacterial translocation. Moreover, the specific reasons and putative mechanisms underlying alterations in gut barrier function in patients with GD have not been explored in this study and require further elucidation.

In conclusion, the current results show that serum levels of leaky gut biomarkers are significantly elevated in patients with initial GD and are associated with TRAb levels, hyperthyroidism, and QOL. Among all assessed biomarkers, zonulin could be useful in distinguishing patients with initial GD from healthy individuals. Moreover, the role of bacterial translocation, particularly regarding increased LPS levels, in the pathogenesis of GD needs to be further investigated in future *in vitro and in vivo* studies.

## Data Availability Statement

The original contributions presented in the study are included in the article/**Supplementary Material**. Further inquiries can be directed to the corresponding authors.

## Ethics Statement

The studies involving human participants were reviewed and approved by the Institutional Review Board of Nanfang Hospital of Southern Medical University. The patients/participants provided their written informed consent to participate in this study.

## Author Contributions

DZ and HL: conceived the study, analyzed data, and wrote the article. SC, XL, ZW, QJ and CZ: collected clinical data. YX, YW, CM and MM: collected clinical samples. YC and LZ: directed the work, and revised the manuscript. All authors contributed to the article and gave final approval of the submitted version.

## Funding

This work was supported by grants from the National Natural Science Foundation of China (No. 82070543 and No.81800460).

## Conflict of Interest

The authors declare that the research was conducted in the absence of any commercial or financial relationships that could be construed as a potential conflict of interest.

## Publisher’s Note

All claims expressed in this article are solely those of the authors and do not necessarily represent those of their affiliated organizations, or those of the publisher, the editors and the reviewers. Any product that may be evaluated in this article, or claim that may be made by its manufacturer, is not guaranteed or endorsed by the publisher.
